# Facial emotion recognition impairment predicts social and emotional problems in children with (subthreshold) ADHD

**DOI:** 10.1007/s00787-020-01709-y

**Published:** 2021-01-07

**Authors:** Anouck I. Staff, Marjolein Luman, Saskia van der Oord, Catharina E. Bergwerff, Barbara J. van den Hoofdakker, Jaap Oosterlaan

**Affiliations:** 1 Department of Clinical, Neuro and Developmental Psychology, Clinical Neuropsychology Section, Vrije Universiteit Amsterdam, Van der Boechorststraat 7-9, 1081 Amsterdam, The Netherlands; 2 Faculty of Psychology and Educational Sciences, KU Leuven, Leuven, Belgium; 3 Department of Developmental Psychology, University of Amsterdam, Amsterdam, The Netherlands; 4 Clinical Neurodevelopmental Sciences, Leiden University, Leiden, The Netherlands; 5 Department of Child and Adolescent Psychiatry, University of Groningen, University Medical Center Groningen, Groningen, The Netherlands; 6 Department of Clinical Psychology and Experimental Psychopathology, University of Groningen, Groningen, The Netherlands; 7 Department of Pediatrics, Amsterdam Reproduction & Development, Emma Children’s Hospital, Amsterdam UMC, University of Amsterdam, Emma Neuroscience Group, Amsterdam, The Netherlands

**Keywords:** ADHD, Facial emotion recognition, Social problems, Emotional problems

## Abstract

Children with attention-deficit/hyperactivity disorder (ADHD) symptoms often experience social and emotional problems. Impaired facial emotion recognition has been suggested as a possible underlying mechanism, although impairments may depend on the type and intensity of emotions. We investigated facial emotion recognition in children with (subthreshold) ADHD and controls using a novel task with children’s faces of emotional expressions varying in type and intensity. We further investigated associations between emotion recognition accuracy and social and emotional problems in the ADHD group. 83 children displaying ADHD symptoms and 30 controls (6–12 years) completed the Morphed Facial Emotion Recognition Task (MFERT). The MFERT assesses emotion recognition accuracy on four emotions using five expression intensity levels. Teachers and parents rated social and emotional problems on the Strengths and Difficulties Questionnaire. Repeated measures analysis of variance revealed that the ADHD group showed poorer emotion recognition accuracy compared to controls across emotions (small effect). The significant group by expression intensity interaction (small effect) showed that the increase in accuracy with increasing expression intensity was smaller in the ADHD group compared to controls. Multiple regression analyses within the ADHD group showed that emotion recognition accuracy was inversely related to social and emotional problems, but not prosocial behavior. Not only children with an ADHD diagnosis, but also children with subthreshold ADHD experience impairments in facial emotion recognition. This impairment is predictive for social and emotional problems, which may suggest that emotion recognition may contribute to the development of social and emotional problems in these children.

**Supplementary Information:**

The online version contains supplementary material available at 10.1007/s00787-020-01709-y.

## Introduction

Attention-deficit/hyperactivity disorder (ADHD) is one of the most common childhood psychiatric disorders. This disorder is characterized by symptoms of inattention, hyperactivity, and/or impulsivity [1]. Although boundaries of the disorder have changed with the different DSM editions [2], approximately 5% of all school-aged children fulfill diagnostic criteria for ADHD [3] and an estimated 11–18% of school-aged children have subthreshold ADHD; these children experience impairments in several domains of functioning because of their ADHD symptoms, although they do not fulfill criteria for a diagnosis [4, 5]. A majority of the children with a full diagnosis of ADHD and subthreshold ADHD experience significant and impairing social and emotional problems [4–6]. Difficulties in social functioning include problems with attuning behavior to others and intrusive behavior [7, 8] which may lead to peer rejection [9], and lack of friendships [10]. Emotional problems include difficulties in the perception of emotions, which is important for adequate social functioning [7] and may increase the risk for anxiety and depression as well as low self-esteem [8]. Studies in children with ADHD confirm that emotional problems can aggravate social problems and increase the risk for comorbid psychopathology [8]. Therefore, it is important to study potential mechanisms that may underlie these socio-emotional problems.

Recent work has focused on emotional dysregulation as a feature of heterogeneity in ADHD [11–14], suggesting that ADHD subtypes may be defined based on emotion regulation profiles. Social impairments in ADHD may be explained by emotional dysregulation [15]. Emotion dysregulation is the inability to modify an emotional state to promote adaptive, goal-directed behavior, and may involve multiple impairments, among which the disability to fully recognize emotional states in facial expressions of others, the disability to modulate the speed and intensity of expressions of both positive and negative emotions (emotional reactivity or lability), and the disability to respond to this emotional reactivity in a way that it is appropriate in the given context and social norms [16, 17]. The current study focused on emotion recognition as a crucial aspect of emotion dysregulation. The ability to encode emotional cues from facial expressions is an important aspect of social functioning [18]. Indeed, impaired facial emotion recognition in children is related to low social competence and lower popularity among peers [19, 20]. Facial emotion recognition impairments have therefore received intensive research in children with ADHD, although results remain mixed [see for reviews: 16,21–24].

To begin with, inconsistent findings refrain us from drawing strong conclusions regarding the degree and type of facial emotion recognition impairments in children with ADHD. A meta-analysis showed that facial emotion recognition abilities in ADHD may depend on the type of emotion studied. In that meta-analysis, controls outperformed children and adults (8–36 years old) with ADHD on facial emotion recognition, albeit effect sizes differed between emotions [24]. For anger (*d* = 0.49) and fear (*d* = 0.48) effects sizes were moderate; for sadness (*d* = 0.24) and happiness (*d* = 0.22) they were small. Another factor that may underlie the inconsistent findings regarding emotion recognition abilities in ADHD is comorbidity with conduct problems: Three recent studies showed no impairment for the recognition of any emotion for children with ADHD compared to controls [25–27], and suggested that the previously reported impairments in emotion recognition may be related to the presence of comorbid conduct problems [22, 26, 28]. Thus, differential effects for type of emotions and/or comorbid problems may explain current inconsistent findings.

Furthermore, the current literature is limited in two aspects. First, studies mainly used high-intensity facial expressions of emotions [24, 27, 29, 30]. Since social interactions often involve very subtle emotional expressions, it is of importance to also study emotion recognition with low-intensity facial expressions of emotions [31]. Studies in typically developing children have indeed shown that the intensity of facial expressions influences recognition ability with higher intensities of adult facial expressions resulting in more accurate emotion recognition [32], although the effect of intensity on accuracy appeared to differ between types of emotions [33, 34]. However, little is known about how the intensity of emotions affects recognition in children with ADHD. One study tested two intensity levels (30% and 70%) of different emotions in a small sample of children with ADHD and controls [35]. Results showed that children with ADHD performed worse than controls for expressions of sadness, while for expressions of anger, group differences only occurred with high-intensity facial expressions. Findings on possible facial emotion recognition impairments may thus depend on expression intensity, although studies using more fine-grained differences in emotional intensity may be needed to fully understand the intensity effect. Finally, most studies showing impairments in facial emotion recognition in children with ADHD compared to controls used pictures of adults [29, 30, 35], while the few studies using children’s faces showed no impairment in children with ADHD [31, 36, 37]. Children might be better in recognizing emotional expressions in the faces of children than in adults, a phenomenon referred to as the own-age bias [38]. Therefore, the emotion recognition impairments in previous studies using adult pictures may not be generalized to children’s pictures, and given that social problems often appear between children, studying effects using children’s faces is of importance and will be done in the current study.

The aim of the current study was to investigate child facial emotion recognition abilities in a group of children with (subthreshold) ADHD (referred to as ADHD group) as compared to typically developing peers, using a newly developed facial emotion recognition paradigm [39, 40]. The paradigm tested facial emotion recognition abilities of the four main emotions (anger, fear, sadness, happiness) each tested at five different intensities (20–100%). As facial emotion recognition impairments have been suggested to underlie social and emotional dysfunctioning in ADHD, we also investigated whether facial emotion recognition abilities were related to real-life difficulties in socio-emotional problems in the ADHD group [16]. Two earlier studies showed that emotion recognition is associated with social skills (i.e., emotion misrecognition is inversely related to social skills) [41], and interpersonal problems (i.e., with parents, teachers, and peers), with the strongest association for anger [35]. As children with subthreshold ADHD experience similar socio-emotional problems compared to children with the full disorder [4], it was hypothesized that the ADHD group would show impairments in emotion recognition compared to controls [16, 21, 24] and that greater problems with emotion recognition would be related to more social and emotional problems in children in the ADHD group [35, 41]. Finally, we explored whether emotion recognition impairments were influenced by the type of emotion, intensity manipulations, and comorbid ODD symptoms.

## Methods

### Participants

The current study included 83 primary school-aged children (6 to 12 years) with (subthreshold) ADHD (referred to as ADHD group), and 30 typically developing classmates as controls (referred to as controls). Children were included in the ADHD group if they: (a) obtained a score > 90th percentile on the teacher-rated Inattention and/or Hyperactivity/Impulsivity scale of the DSM-based Disruptive Behavior Disorders Rating Scale (DBDRS) [42, 43]; (b) showed at least three symptoms (item score ≥ 2) on the Inattention and/or Hyperactivity/Impulsivity scale of the semi-structured DSM-IV-TR Teacher Telephone Interview (TTI) [44]; and (c) obtained a score > 5 (indicating impairment, range 0–10) on at least one domain functioning of the teacher-rated Impairment Rating Scale (IRS) [45]. For five children inclusion took place before the summer holiday. For these children, impairment was based on the fact that the teacher was seeking help to cope with the child’s behavior, which was substantiated by TTI scores. To avoid overlap between the ADHD and control groups (see below), children within the ADHD group who scored < 80th percentile on the Hyperactivity scale of the teacher-rated Strengths and Difficulties Questionnaire (SDQ) were excluded [46, 47] for this study.

The typically developing control children were classmates of the children assigned to the ADHD group, and were recruited through the teachers that could nominate a classmate of the same age. Controls were required not to attend special education classes and to obtain a score < 80th percentile on the Hyperactivity scale of the teacher-rated SDQ [46, 47].

Children were excluded if they: (a) had an estimated full-scale IQ lower than 70 as assessed with a short form of the Wechsler Intelligence Scale for Children-third edition (WISC-III) [48] including the subtests Block Design and Vocabulary [49], (b) were taking psychotropic medication during the last month, or (c) had a diagnosis of autism spectrum disorder or conduct disorder according to the DSM-IV-TR [50] or DSM-5 [1] as reported by parents.

See Table 1 for group characteristics.

**Table 1:** Sample characteristics

	ADHD (*n* = 83)	Control (*n* = 30)	Group comparisons
Age at assessment in years, *M* (SD)	8.34 (1.64)	8.20 (1.46)	*t*(111) = .41, *p* = .683, *d* = .09
Sex, *n* (%) boys	68 (82)	13 (43)	*χ*^2^ = 16.17, *p* < .001, *V* = .38
IQ, *M* (SD)	100.55 (12.23)	105.20 (15.21)	*t*(111) = − 1.67, *p* = .098, *d* = .36
TTI symptom severity, *M* (SD)			
Inattention	4.53 (1.82)		
Hyperactivity/impulsivity	4.20 (2.28)		
ODD	1.10 (1.45)		
CD	.05 (.31)		
*Teacher ratings*			
SDQ, *M* (SD)			
Hyperactivity	8.65 (1.27)	.57 (.82)	*t*(80.32) = 39.55,* p* < .001, *d* = 6.91
Emotional problems	2.30 (2.32)	0.87 (1.41)	*t*(84.95) = 3.97, *p* < .001, *d* = .67
Peer problems	2.31 (2.25)	0.63 (1.00)	*t*(106.28) = 5.48, *p* < .001, *d* = .84
Prosocial behavior	6.06 (2.31)	9.13 (1.20)	*t*(97.28) = − 9.19, *p* < .001, *d* = 1.48
DBDRS, *M* (SD)			
Inattention	16.92 (4.84)		
Hyperactivity/impulsivity	15.60 (6.71)		
ODD	7.27 (5.73)	0.53 (.97)	*t*(93.91) = 10.30, *p* < .001, *d* = 1.36
IRS, *M* (SD)			
Number of domains impairment	3.90 (1.18)		
SWAN, *M* (SD)			
Inattention	13.98 (5.67)	− 12.23 (7.36)	*t*(50.51) = 16.14, *p* < .001, *d* = 4.26
Hyperactivity/impulsivity	13.90 (7.50)	− 12.27 (7.65)	*t*(111) = 16.29, *p* < .001, *d* = 3.47
*Parent ratings*			
SDQ, *M* (SD)^a^			
Hyperactivity	6.83 (2.38)	1.34 (1.84)	*t*(63.28) = 12.74,* p* < .001, *d* = 2.44
Emotional problems	2.54 (2.40)	1.76 (1.66)	*t*(71.15) = 1.91, *p* = .060, *d* = .35
Peer problems	1.70 (1.50)	0.66 (0.81)	*t*(90.75) = 4.63, *p* < .001, *d* = .77
Prosocial behavior	8.50 (1.39)	8.86 (1.68)	*t*(109) = − 1.14, *p* = .257, *d* = .24
DBDRS, *M* (SD)^a^			
ODD	5.78 (4.36)	2.38 (1.99)	*t*(102.06) = 5.61 *p* < .001, *d* = .88
SWAN, *M* (SD)^b^			
Inattention	6.70 (7.37)	− 9.45 (8.04)	*t*(104) = 9.81, *p* < .001, *d* = 2.14
Hyperactivity/impulsivity	8.40 (6.99)	− 11.97 (9.05)	*t*(41.26) = 10.96, *p* < .001, *d* = 2.69

*ADHD* attention-deficit/hyperactivity disorder, *DBDRS* Disruptive Behavior Disorders Rating Scale, *IRS* impairment rating scale, *SDQ* Strengths and Difficulties Questionnaire, *SWAN* strengths and weaknesses of attention-deficit/hyperactivity symptoms and normal behaviors, *TTI* teacher telephone interview

^a^Two parents did not complete this questionnaire (one in the ADHD group, one in the control group)

^b^Seven parents did not complete this questionnaire (six in the ADHD group, one in the control group)

### Materials

#### Behavioral questionnaires

*Inattention and hyperactivity/impulsivity*. To check whether our allocation to groups was successful, parents and teachers completed the Strengths and Weaknesses of ADHD-symptoms and Normal Behavior (SWAN) rating scale [51]. This rating scale contains two scales measuring inattention (nine items) and hyperactivity/impulsivity (nine items), on which a child’s behavior compared to peers is rated on a 7-point Likert scale. All scores were reverse scored for consistency with other measures used in this study. The resulting scores may range between − 27 and 27 for both scales, with higher scores indicating more ADHD symptoms. The internal consistency for the SWAN is high (*α* = 0.95), and convergent and discriminant validity has been established [51].

*Oppositional behavior*. Symptoms of Oppositional Defiant Disorder (ODD) [1] were measured with the ODD-scale of the DBDRS [42, 43] administered to both parents and teachers of the child. The ODD scale consists of eight items, rated on a 4-point Likert scale (0 = *not at all*, 3 = *very much*). Raw scores may range between 0 and 24, with higher scores indicating more symptoms. The internal consistency is high (*α* = 0.95), and convergent validity is strong [42].

*Socio-emotional problems.* Social and emotional functioning was assessed using both a parent and teacher-rated SDQ [47, 52]. This rating scale consists of 25 items on a 3-point Likert scale. The items are divided into five scales of five items each (scores may range from 0 to 10 per scale). For this study, we used the following scales to assess social and emotional functioning: Emotional Problems, Peer Problems, and Prosocial Behavior. The Dutch version of the SDQ has adequate psychometric properties [46, 53].

#### Emotion recognition task

**Fig. 1:**
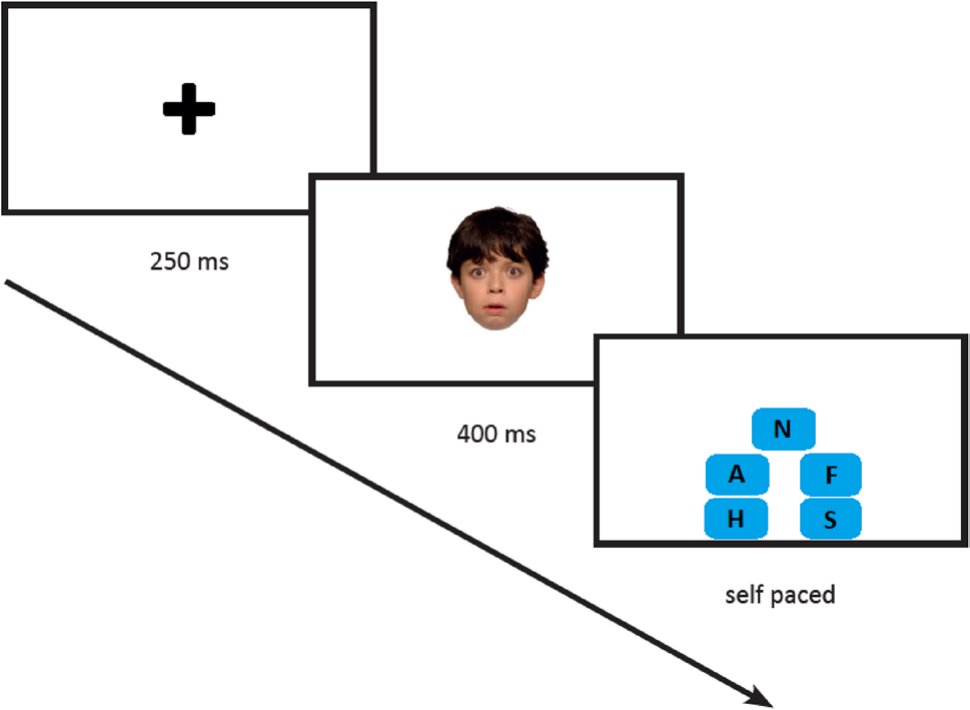
Course of a trial. Abbreviations in the text boxes in the third screen represent the emotion labels anger (A), fearful (F), happy (H), sad (S) and neutral (N). Emotion labels were written out completely in the actual task [39]

A modified version of the Morphed Facial Emotion Recognition Task (MFERT) was used to assess facial emotion recognition [39, 40]. Pictures of the faces of six different child actors (three males, three females), displaying high-intensity expressions (100%) of anger, fear, sadness, and happiness, and a neutral expression (0%) were used. We selected four emotions of which the recognition of expressions develop first in life, given the age range of our sample [54, 55]. The pictures were taken from the validated NIMH Child Emotional Faces Picture Set (NIMH-ChEFS). Inter-rater agreement regarding the expressed emotions in these pictures was > 0.80 [56]. Using the Abrosoft FantaMorph software (Abrosoft, USA), the neutral expression of a child actor was morphed with the high-intensity expression for each emotion, with steps of 20% increments. The resulting stimuli varied in emotional intensity from 20% (80% neutral) to 100% (0% neutral) (see Supplementary Material S1 for an example of emotional expressions in varying intensity levels). The 126 pictures (four emotional conditions by five intensity levels, and a neutral expression, expressed by six actors) were presented in random order using E-prime, version 2.0 [57]. Task design is depicted in Fig. 1. Each trial started with a fixation cross that was displayed for 250 ms, followed by the stimulus that was presented for 400 ms. Given that emotional expressions have to be identified quickly in daily life social interactions [58], and that having a longer stimulus presentation or a self-paced paradigm may result in ceiling effects [32, 33], stimulus presentation was limited to 400 ms. It is shown that conscious emotion processing in children occurs within this interval [59]. The response options (anger, fear, sadness, happiness, neutral) remained visible on the screen throughout the task. Participants had to indicate the emotion corresponding to the target stimulus by clicking on one of the five emotion labels with a computer mouse. The 126 test trials were presented in two blocks of 63 trials, with a small break in between. The test blocks were preceded by 30 practice trials, consisting of trials with the four high-intensity expressions (100%) and the neutral expression (0%) of six child actors. These actors were three males and three females, and were different than the actors used for the test blocks. Outcome variables were accuracy scores (percentage correct) and reaction times (RT) in ms per expression intensity level per emotion condition [see 39]. Trials with extreme slow responses (reaction time > 3 SDs above the child’s mean) were excluded for exploratory (see below) RT analyses (excluding less than 2% of the data).

### Procedure

The medical ethical committee of the University Medical Center Groningen (UMCG) approved the study procedure. The study was conducted in accordance with the Declaration of Helsinki (2013). Teachers were recruited through school principals, educational consult associations, and outpatient mental health clinics. Teachers, parents, and children older than 11 years, provided consent. Teacher and parent questionnaires were administered via a secured website. Interviews with teachers were administered by the first author (AS). The WISC-III short form and MFERT (in this order) were administered in a quiet room at the child’s school. Standardized instructions were used for the MFERT. In line with the protocol approved by the medical ethical committee, parents of participating children were debriefed about the aim of the study after completing the tasks.

### Data analysis

Statistical analyses were performed using SPSS IBM, version 25. Differences in demographic characteristics, IQ and ADHD related symptoms and impairment between the ADHD group and controls were assessed using independent samples *t*-tests and chi-square test. Missing values were not imputed. Differences in emotion recognition accuracy were tested using a repeated-measures ANOVA, with group (ADHD, control) as between-subject factor, and emotion condition (happy, anger, fear, sad) and expression intensity (5 morphed levels; 20%, 40%, 60%, 80%, 100%) as within-subject factors. The effects of expression intensity on accuracy were tested using a polynomial trend analysis. When interactions involving emotion condition or expression intensity were significant, post hoc analyses were performed investigating effects of emotion condition and/or expression intensity level. Greenhouse–Geisser correction was applied if the assumption of sphericity was violated. Effect sizes were expressed in terms of $\eta_{p}^{2}$, with 0.01, 0.06, and 0.14 referring to small, medium, and large effects, respectively [60]. To explore whether group differences were driven by systematic errors committed to interpreting a specific emotional condition (i.e., whether there was evidence for emotional recognition bias for one of the type of emotions) [22, 61], ADHD and controls were compared on the type of errors committed (i.e., interpreting an emotion condition erroneously as neutral, happiness, anger, fear or sadness, collapsed across expression intensity levels), using repeated-measures ANOVA. Finally, to explore whether (possible) group differences in accuracy were related to differences in speed-accuracy tradeoff of recognizing emotional expressions (i.e., whether more accurate responders were those who responded slower), we examined RTs for correct trials using repeated-measures ANOVA. Analyses were performed similar to those on the accuracy, although now RT rather than percentage correct served as an outcome. Given that most children were not able to successfully distinguish between emotions presented at low-intensity levels, these data did not allow us to study the effects of intensity level in the analyses on RT, and thus this analyses only tested the effects of group and the group by emotion type interaction.

Exploratory analyses compared children with low and high levels of ODD symptoms in the ADHD group to examine whether comorbid ODD symptoms affected emotion recognition in these children. Children in the ADHD group were allocated to the low and high on ODD group based on median split analysis, according to teacher ratings on the ODD-scale of the DBDRS. The groups were compared on accuracy, committed errors, and RT on accurate trials. We also did a sensitivity analysis, comparing those children in the ADHD group that had not received a clinical diagnosis of ADHD according to parental report to the control group, to check whether (possible) group differences in accuracy held up.

Multiple regression analyses examined whether emotion recognition abilities predicted social and emotional problems (parent- and teacher ratings on the SDQ scales Peer Problems, Emotional Problems and Prosocial Behavior) within the ADHD group. To measure socio-emotional problems across settings, to enhance reliability, and to reduce error variance, for each of the three SDQ scales, parent and teacher ratings were averaged. To adjust for the effects of sex (boy = 0, girl = 1) and age (months), these variables were entered as predictors in the first step. Percentage correct across all emotion conditions and intensity levels was added in the second step. Alpha level was adjusted for the separate regression analyses on three SDQ scales (0.05/3).

### Power analysis

We conducted power analysis using G*power to determine the number of subjects needed to test for group differences (ADHD versus control) and group by emotion condition or group by expression intensity level interactions, using repeated measures with 21 measurements (4 emotions × 5 expression intensities + 1 neutral condition). We expected a small to medium group difference, given the inconsistencies in previous studies regarding impairments in emotion recognition of children with ADHD (Cohen’s *f* = 0.20). Power analysis revealed that we needed a total of 68 children to have sufficient power to detect group differences, and 20 children to detect a group by emotion condition or expression intensity level interactions (alpha = 0.05, power = 0.80). To detect a three-way interaction effect, roughly fourfold of the sample size required for a two-way interaction effect is required (i.e., 80 children for the current study) [62].

## Results

Group comparisons revealed no group differences in age and IQ. There were more boys in the ADHD group than in the control group (see Table 1). Therefore sensitivity analyses were run for all main analyses with groups matched on sex and age. Supporting the validity of our inclusion procedures, children in the ADHD group showed higher teacher and parent ratings of ADHD symptoms, oppositional defiant behavior, and peer problems, compared to controls (medium to large effects). Furthermore, the ADHD group showed higher teacher-rated emotional problems and less teacher-rated prosocial behavior (small to medium effect).

Parents reported that a total of 24 of 83 children in the ADHD group (29%) had received a clinical diagnosis of ADHD as established by a child psychiatrist or pediatrician, and that three children (4%) had been diagnosed with a comorbid learning disorder. None of the children had a clinical diagnosis of ODD or were on pharmacological treatment for ADHD. In the control group, one child (1%) had a language disorder, none had a diagnosis of ADHD or ODD.

### Group comparisons

Since unequal sample sizes may affect ANOVA results by violating the assumption of homogeneity of variances, we tested whether both groups had equal variances on all 21 outcomes. Levene’s test showed that variances were similar on 16 out of 21 outcomes (*p* = 0.051—0.929). For the five outcomes on which Levene’s test was significant, variances were larger in the ADHD group, indicating that the F-statistic is conservative and that violating this assumption for these outcomes will not be problematic [63].

**Fig. 2:**
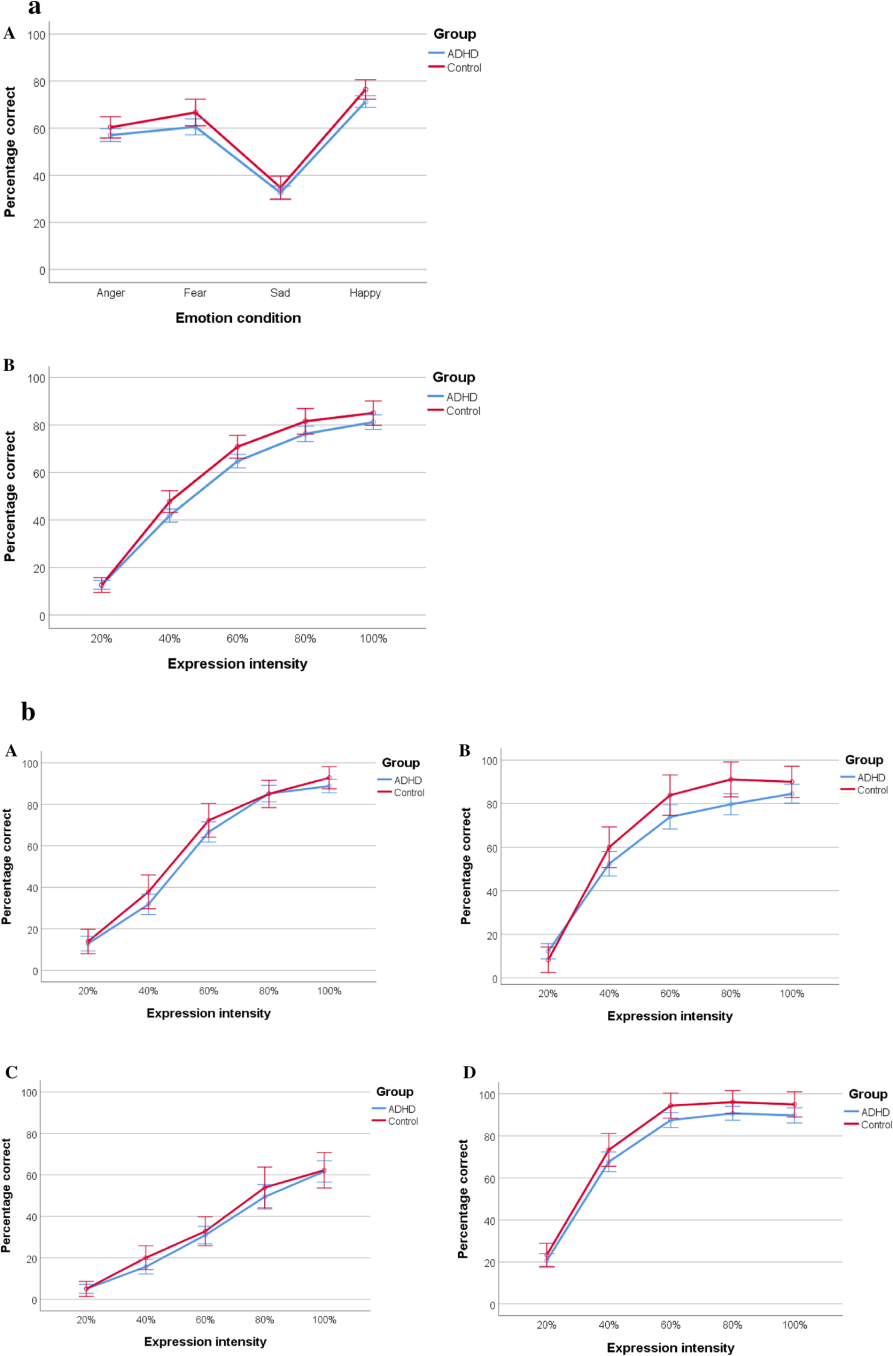
**a** Line graphs of the effects of emotion condition (panel A) and expression intensity (panel B) on emotion recognition accuracy. **b** Line graphs of the effects of expression intensity on the recognition of anger (panel A), fearful (panel B), sad (panel C), and happy expressions (panel D). Error bars reflect 95% confidence intervals

Figure 2a demonstrates that children in the ADHD group showed poorer emotion recognition abilities compared to controls, as confirmed by a small-sized main effect of group on accuracy (*F*(1, 111) = 4.32, *p* = 0.040, $\eta_{p}^{2}$ = 0.04). There was also the main effect of emotion condition (*F*(3, 2.709) = 221.87, *p* < 0.001, $\eta_{p}^{2}$ = 0.67), indicating that accuracy differed between emotions. Expressions of happiness (*M* = 73.87, SD = 1.22) were recognized most accurately, followed by expressions of fear (*M* = 63.61, SD = 1.67), anger (*M* = 58.68, *SD* = 1.33), and sadness (*M* = 33.65, SD = 1.42) (all *p*-values < 0.001, except anger vs. fear: *p* = 0.006). The effects of emotion condition on accuracy did not differ between the groups, confirmed by a non-significant group by emotion condition interaction on accuracy (*F*(3, 2.709) = 0.58, *p* = 0.611, $\eta_{p}^{2}$ < 0.01). Thus, children in the ADHD group performed worse compared to controls, independent of emotional condition.

For expression intensity, the main effect on accuracy was found (*F*(4, 2.705) = 794.57, *p* < 0.001, $\eta_{p}^{2}$ = 0.88), with the linear trend showing the best fit ($\eta_{p}^{2}$ = 0.93), indicating that accuracy increased with increasing expression intensity. Also, the quadratic effect was significant ($\eta_{p}^{2}$ = 0.78) and the quadratic effect of expression intensity differed between groups, as indicated by a significant group by intensity interaction (*F*(1, 111) = 5.27, *p* = 0.024, $\eta_{p}^{2}$ = 0.05). Figure 2b shows that accuracy slopes were blunted for the ADHD group compared to controls with conversion in the high-intensity emotions.

The three-way interaction between group, emotion condition, and expression intensity was not significant (*F*(12, 9.611) = 0.80, *p* = 0.625, $\eta_{p}^{2}$ < 0.01), indicating that the group by expression intensity interaction did not differ between emotional conditions.

Table 2 shows the type of committed errors (collapsed across emotion conditions and expression intensity levels) for the ADHD and control groups. The main effect of type of error was significant (*F*(4, 1.412) = 361.84, *p* < 0.001, $\eta_{p}^{2}$ = 0.77), indicating that the frequency of errors differed between types of errors. Across both groups, children more often erroneously choose for a neutral expression than for expressions of sadness, anger, fear, and happiness (neutral versus other: *F*(1, 111) = 435.01, *p* < 0.001, $\eta_{p}^{2}$ = 0.80), suggesting that facial expressions are often not recognized as an emotional expression. This effect was largest for sadness (*M* = 12.79, SD = 4.59), followed by anger (*M* = 10.12, SD = 4.25), fear (*M* = 8.22, SD = 3.73) and happiness (*M* = 6.09, SD = 3.20) (all *p*-values < 0.001). The group by error type interaction was not significant (*F*(4, 1.412) = 0.27, *p* = 0.685, $\eta_{p}^{2}$ < 0.01), showing that groups did not differ in the type of errors committed.

**Table 2:** Error rates in the five emotion conditions

Emotion condition erroneously interpreted as	ADHD, *M* (SD)	Control, *M* (SD)	Group comparisons
Neutral	37.82 (13.17)	35.60 (12.83)	*t*(111) = .80, *p* = .427, *d* = .17
Happy	3.12 (3.34)	2.30 (3.29)	*t*(111) = 1.14, *p* = .256, *d* = .25
Anger	5.11 (4.42)	5.13 (5.03)	*t*(111) = − .03, *p* = .980, *d* < .001
Sad	3.19 (3.51)	2.13 (2.30)	*t*(78.59) = 1.86, *p* = .067, *d* = .33
Fear	5.76 (4.78)	4.67 (5.27)	*t*(111) = 1.04, *p* = .299, *d* = .22

*ADHD* attention-deficit/hyperactivity disorder

Finally, it was tested whether groups differed on RT of correct trials. Repeated measures ANOVA did not reveal an effect of group on RT (*F*(1, 111) = 0.67, *p* = 0.416, $\eta_{p}^{2}$ = 0.01), nor an interaction between group and emotion condition (*F*(2.669, 296.24) = 0.81, *p* = 0.475, $\eta_{p}^{2}$ =0.01). For incorrect trials, the effects of group and group by emotion were also non-significant.

### Sensitivity analyses

To check whether the observed group differences in the sex distribution affected the results, we matched the ADHD and control group on sex and age. For accuracy, results remained unchanged for the main effect of group (*F*(1, 54) = 6.86, *p* = 0.011, $\eta_{p}^{2}$ = 0.11), and the non-significant group by emotion condition interaction (*F*(3, 2.769) = 0.47, *p* = 0.469, $\eta_{p}^{2}$ = 0.02). With regard to the group by intensity interaction, now the linear trend (i.e., rather than the quadratic trend) showed the largest percentage of variance explained, although this effect just escaped conventional levels of statistical significance (*F*(1, 54) = 3.60, *p* = 0.063, $\eta_{p}^{2}$= 0.06). Further, sex was not related to emotion recognition accuracy (*r* = 0.02, *p* = 0.832), and boys (*M* = 60.51, SD = 9.34) and girls (*M* = 60.92, SD = 9.54) did not differ in their accuracy on the MFERT (*t*(111) = -0.21, *p* = 0.832).

We explored whether comorbid ODD symptoms as assessed with the teacher-rated DBDRS affected the results. Because of the skewed distribution of these scores, median split analyses (median = 6) were used to compare children with low (score < 6, *N* = 38) and high levels of ODD symptoms (score > 6, *N* = 39) in the ADHD group. Children with low and high levels of ODD symptoms did not differ on accuracy: the main effect of ODD group (*F*(1, 75) = 0.29, *p* = 0.593, $\eta_{p}^{2}$ = 0.00), ODD group by emotion condition interaction (*F*(2.64, 197.89) = 1.60, *p* = 0.197, $\eta_{p}^{2}$ = 0.02), ODD group by intensity interaction (*F*(2.59, 193.97) = 0.73, *p* = 0.516, $\eta_{p}^{2}$ = 0.01), and the ODD group by emotion condition by intensity interaction (*F*(9.11, 683.12) = 0.90, *p* = 0.531, $\eta_{p}^{2}$ = 0.01) were all non-significant. Further, ODD groups did not differ in the types of errors committed (*F*(3.06, 226.62) = 0.886, *p* = 0.463, $\eta_{p}^{2}$ = 0.01). There was a main effect of ODD symptoms on RT (*F*(1,75) = 5.54, *p* = 0.021, $\eta_{p}^{2}$ = 0.07), showing that the children with high levels of ODD responded faster on correct trials than the children with low levels of ODD. This effect did not differ between types of emotions, as indicated by a non-significant group by emotion condition interaction: *F*(2.81,210.51) = 0.32, *p* = 0.796, $\eta_{p}^{2}$ < 0.01.

Finally, we explored whether results on accuracy held up when comparing children in the ADHD group of whom parents did not report a clinical diagnosis (*N* = 59) to the control group (*N* = 30). Results showed that all findings were essentially replicated with similar effect sizes. The group by intensity interaction remained significant with the largest percentage of variance explained by the quadratic trend (*F*(1, 87) = 5.98, *p* = 0.016, $\eta_{p}^{2}$ = 0.06), although the main effect of the group just escaped conventional levels of statistical significance (*F*(1, 87) = 2.90, *p* = 0.092, $\eta_{p}^{2}$ = 0.03).

### Multiple regression analyses

We used accuracy of emotion recognition, regardless of emotion and intensity, as a predictor in the regression analyses in the ADHD group. After adjusting for sex and age, accuracy contributed a significant 7% of the variance to the aggregated parent and teacher-rated emotional problems on the SDQ (Δ*R*^2^ = 0.07, *p* = 0.012). Higher overall accuracy scores were associated with less emotional problems. For aggregated parent and teacher ratings of peer problems, a similar pattern emerged: after adjusting for sex and age, accuracy contributed a significant 14% of the variance to peer problems (Δ*R*^2^ = 0.14, *p* < 0.001). Higher overall accuracy scores were associated to lower peer problems. Accuracy scores did not significantly contribute to the prediction of ratings of prosocial behavior.

## Discussion

The aim of the current study was to examine facial emotion recognition in children with (subthreshold) ADHD, as compared to typically developing controls, using a novel task in which the intensity of different types of emotional expressions was manipulated. Furthermore, we examined whether impairment in emotion recognition accuracy was related to social and emotional problems in the ADHD group. In line with our hypothesis, results showed that the ADHD group performed worse than controls across types and intensities of emotional expressions (small effect), with a smaller increase in accuracy with increasing intensities as compared to control children (small effect). Despite the small group differences, it appeared that the severity of emotion recognition problems was a predictor of the severity of emotional and peer problems in the ADHD group.

In this study, we used a newly developed task to assess facial emotion recognition in children, using four emotions on five intensity levels of child facial emotional expressions. This allowed us to examine subtle differences in facial emotion recognition abilities between children with (subthreshold) ADHD and controls. The main effects of emotion condition and expression intensity found on accuracy support the validity of the task. Finally, we presented facial expressions for a short time increasing its ecological validity [58].

Our study showed that children with (subthreshold) ADHD experience mild problems with facial emotion recognition, regardless of the type of emotion. Group differences were smaller compared to some previous studies in clinical ADHD samples [16, 21], although other studies in clinical ADHD samples failed to observe any group differences in facial emotion recognition [25–27]. Our results contribute to the literature by showing that children with subthreshold ADHD experience highly similar problems compared to those with a full diagnosis [5]. Further, and in contrast to the bulk of literature on emotion recognition, we used child facial expressions instead of adult faces as stimuli, and showed small-sized group differences, compared to medium-sized group differences observed in some of the previous studies using adult faces [see for reviews: 16,23]. This may suggest that recognizing facial emotional expressions is easier with children’s faces compared to adult faces [64]. Those previous studies that did use children’s faces did not find evidence for impaired facial emotion recognition in children with ADHD [31, 36, 37]. These conflicting results might be related to the use of high-intensity facial expressions only in the previous studies, which are likely more easy to recognize for children, obscuring more subtle differences between children with and without ADHD. Future studies directly comparing child and adult facial emotion expressions should be set up to conclude on whether facial expressions are easier to recognize for children when expressed by their same-age peers.

The ADHD group showed a smaller increase in their facial emotion recognition abilities with increasing intensities compared to controls with differences attenuating with high intensities. This indicates that children with (subthreshold) ADHD have particular difficulty in recognizing emotions when expressions are more subtle. In our sensitivity analyses in the sex (and age) matched groups, the group by intensity interaction was no longer significant (although controls still outperformed the ADHD group). It seems unlikely that sex affected the obtained overall results given that sex was not related to accuracy and boys and girls (collapsed across both groups) showed similar task performance. This non-significant result is likely to be explained by a reduction of power in sensitivity analyses.

Previous studies suggested that behavior disorder problems (e.g., symptoms of oppositional and conduct problems) may in fact account for the impairment in facial emotion recognition in children with ADHD [22, 26, 28]. Our findings do not confirm this hypothesis, given that the emotion recognition accuracy of children in the ADHD group was independent of levels of ODD symptoms, and the number of conduct disorder symptoms was overall low in our group. However, children with low levels of ODD symptoms responded slower on correct trials than children with high levels of ODD symptoms, which leaves open the suggestion that children with high levels of ODD symptoms have less emotion recognition impairments compared to children with low levels of ODD symptoms (i.e., faster responses while accuracy is similar compared to children with low levels of ODD symptoms). Therefore, further research into the role of comorbid behavioral problems in explaining the emotion recognition disabilities of children with ADHD is warranted. Furthermore, it appeared that children in the ADHD group showed the same error pattern as controls. This suggests that impairments in facial emotion recognition do not reflect a biased perception of emotions as seen in samples with comorbid conduct problems (i.e., emotion recognition bias) [61, 65, 66].

To relate task performance to real-life functioning, we investigated whether impaired facial emotion recognition in the ADHD group was related to social and emotional problems in this group. Indeed, emotion recognition accuracy was inversely related to social and emotional problems in the ADHD group. Apparently, difficulties with the attribution of the correct emotional state to a facial emotional expression are related to peer problems. Children with ADHD are often described as impulsive and intrusive in their social behavior [7], and our results suggest that this may be related to difficulties encoding someone’s emotional state. These difficulties may in the end lead to further problems in social-cognitive information processing, such as interpreting behavior and in choosing an adequate behavioral response, and eventually making and keeping friends. Further, our results show that emotion recognition problems are associated with increased emotional problems such as feeling sad, worried or afraid. However, the causal pathway of this relation may be bi-directional: whereas internalizing problems could lead to impaired emotion recognition [67–69], emotion recognition problems may also lead to aggravated peer problems and increased internalizing problems [70]. Since children with emotion recognition impairments have recently been detected as separate ADHD subgroup based on neurocognitive profiles [71], it seems relevant to assess emotion recognition as part of clinical assessment. Children with emotion recognition impairments might benefit from interventions aimed at improving social functioning in ADHD (e.g., social skills training programs). Such interventions often include elements of emotion awareness, such as emotion recognition [72]. The current findings emphasize the importance of focusing on improving emotion recognition skills in those interventions.

Despite clear strengths, the current study also has some limitations. First, the sample of children in the ADHD group was recruited through teachers and inclusion criteria were based on teacher measures only. The severity of ADHD symptoms and impairment might therefore be less than in children with a full diagnosis of ADHD. However, teachers reported at least subthreshold levels of ADHD symptoms at school, one-third of the children had a clinical diagnosis of ADHD, and almost the whole sample obtained parent ratings of ADHD in the clinical range, indicating that these children had substantial ADHD symptoms and related impairment. Second, we did not take comorbid autism spectrum disorder (ASD) symptoms into account, although a clinical diagnosis of ASD as indicated by parents was an exclusion criterion for this study. Given the high comorbidity rates for ADHD with ASD [73] and similarities between impairments in emotion recognition abilities in both disorders [74], results could have been affected by comorbid ASD symptoms. Third, because we did not include a non-emotional control task, we cannot rule out the contribution of other neurocognitive impairments on task performance. Some authors have argued that impaired facial emotion recognition may be explained by neurocognitive weaknesses [27], such as the inability to attend to relevant cues of affect (sustained attention), the failure to inhibit impulsive responses (inhibition) or impairments in integrating multiple sets of verbal and nonverbal communication (working memory). Future studies may investigate whether emotion recognition impairments in ADHD may be explained by neurocognitive functioning.

To conclude, this study used a newly developed task and showed there were case–control differences with small effect sizes in emotion recognition accuracy across emotions for children with (subthreshold) ADHD as compared to controls, with the ADHD group showing a smaller increase in accuracy with increasing emotion intensities. Effects were unrelated to comorbid ODD and sex. Severity of emotion recognition impairments was related to social and emotional problems in children with (subthreshold) ADHD, and may reveal one possible mechanism to explain socio-emotional problems in this group. Future longitudinal studies are needed to examine whether facial emotion recognition impairments play a role in the development of socio-emotional problems in ADHD and whether facial emotion recognition abilities and the proposed resulting social and emotional problems, could be improved by interventions [72, 75].

## Electronic supplementary material

Below is the link to the electronic supplementary material.

Supplementary file1 (DOCX 308 kb)

## Data Availability

The data that support the findings of this study are available from the corresponding author (AS), upon reasonable request.
