# Supplementary figures and images for: Facial emotion recognition impairment predicts social and emotional problems in children with (subthreshold) ADHD
Source: Eur Child Adolesc Psychiatry. 2021 Jan 7;31(5):715–27. doi: 10.1007/s00787-020-01709-y (PMC9142461; doi:10.1007/s00787-020-01709-y)

**Supplementary Material**

| 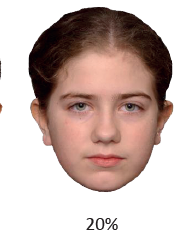 | 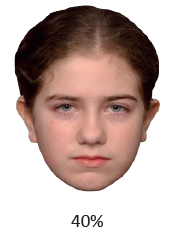 | 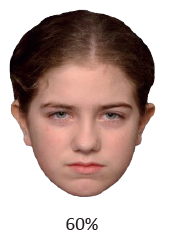 |
| --- | --- | --- |
| 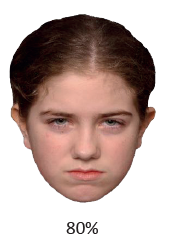 | 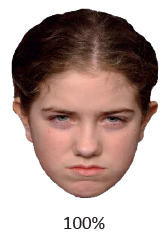 |  |
| *Fig*. S1. Examples of angry expressions at varying intensity levels | | |

Supplement: Supplementary file 1 — Supplementary file1 (DOCX 308 kb) [file 787_2020_1709_MOESM1_ESM.docx]
